# Antibacterial potential of endophytic *Streptomyces* spp. isolated from peanut (*Arachis hypogaea*) roots: bioactiveprofiling and molecular docking studies

**DOI:** 10.1038/s41598-026-36976-3

**Published:** 2026-02-10

**Authors:** Rehab M. Mohamed, Mohamed E. El Awady, Asmaa M. Fahim, Aly E. Abo-Amer

**Affiliations:** 1 Department of Botany and Microbiology, Faculty of Science, Sohag University, Sohag, 82524 Egypt; 2 Microbial Biotechnology Department, Biotechnology Research Institute, National Research Centre, Cairo, Egypt; 3 Department of Green Chemistry, National Research Centre, P.O. Box 12622, Dokki, Cairo, Egypt

**Keywords:** *Arachis Hypogaea* (peanut), Endophytes, *Streptomyces* spp., Bioactive compounds, Antimicrobials, Docking and molecular dynamics, Biochemistry, Biotechnology, Microbiology

## Abstract

The worldwide escalation of antimicrobial resistance (AMR) necessitates the search for new bioactive agents from natural sources. Consequently, this study investigates the antimicrobial activity of endophytic *Streptomyces* spp. Eighteen *Streptomyces* isolates were recovered from sixteen peanut root samples using nitrate starch agar at 30 °C for 7 days. Among these, two strains, *Streptomyces rochei* RSA1 and *Streptomyces* sp. RSA2, exhibited significant antibacterial activity against both Gram-positive (*Bacillus cereus* and *Staphylococcus aureus*) and Gram-negative (*Pseudomonas aeruginosa* and *Escherichia coli*) bacteria, compared with six standard antibiotics. Nine metabolic bioactive compounds were identified using GC-MS. However, two compounds2-(butylthio) pyrimidine-4,6(1 H,5 H)-dione and 2,4-di-tert-butylphenol were particularly prominent (> 96% abundance). Functional groups were confirmed via FT-IR spectra. Molecular docking and dynamics simulations with relevant bacterial protein targets (PDB ID: 6FJH, 1O9G, 1J5E, and 9QT5) revealed strong hydrogen bonding and electrostatic interactions, with *S. rochei* RSA1 forming the most stable complex. Overall, peanut-derived endophytic *Streptomyces* represent promising sources of bioactive antibacterial metabolites for combating multidrug-resistant infections.

## Introduction

 Antimicrobial resistance (AMR) has emerged as one of the most pressing global public health threats of the 21 st century. The World Health Organization (WHO) has warned that AMR is currently responsible for 700,000 deaths annually, and it is estimated that by 2050 this figure could rise to 20 million^1^. The emergence of drug-resistant bacteria has made the treatment of infections in healthcare settings increasingly difficult, leading to a rise in hospital-acquired infections. The persistent surge of antibiotic-resistant bacteria now represents one of the gravest challenges to global health, undermining the efficacy of frontline treatments and driving an urgent search for new therapeutics^1^. AMR is increasing worldwide, with bacterial infections remaining a primary cause of morbidity and mortality. Novel resistance mechanisms frequently emerge and disseminate globally, making AMR a substantial concern due to its potential for international spread and the consequent limitations in treatment options^2^.

Endophytes, a group of microorganisms, mostly bacteria or fungi can colonize inter- and/or intracellular parts of plants without causing apparent disease^3^. While the majority of endophytes exert a neutral effect on plants, some confer beneficial effects and have recently gained attention for sustainable agricultural practices aimed at minimizing losses and enhancing crop productivity^4^. The molecular mechanisms underlying endophytic colonization, however, are not yet fully understood^5^.

Endophytic bacteria, commonly referred to as plant growth-promoting rhizobacteria (PGPR), are a subclass of rhizospheric bacteria that share key traits associated with host plant growth promotion^5^. They also synthesize bioactive compounds to compete for resources and protect against diseases. These compounds can inhibit the proliferation of competing microorganisms, enhance plant defenses, promote symbiotic associations, and ultimately support the survival of both the bacteria and their host plants^6^. Literature indicates that endophytic associations drive the evolution of biosynthetic gene clusters responsible for secondary metabolite production, making them significant sources for drug discovery and development. Metabolites produced by endophytic *Streptomyces* exhibit anticancer, antifungal, antibacterial, insecticidal, and other antimicrobial properties^7^.

Natural products, particularly those produced by the hidden inhabitants of plants, have emerged as a fertile frontier in the search for novel therapeutics. Among endophytes, *Streptomyces* spp., a distinguished subgroup of Actinobacteria, stand out for their rich repertoire of secondary metabolites, including antifungals, antibiotics, and anticancer agents. Through the secretion of phytohormones, enhancement of nutrient acquisition, and deployment of antibiosis and systemic resistance, these *Streptomyces* establish mutually beneficial relationships with their host plants-partnerships that have, to date, provided over half of the antibiotics used in clinical practice^8,9^.

Yet, many endophytic *Streptomyces* from understudied hosts remain unexplored. Peanut (*Arachis hypogaea*) roots, with their complex rhizosphere microbiomes, may harbor novel *Streptomyces* strains capable of synthesizing previously unidentified antibacterial compounds. Although earlier studies have demonstrated the potency of *Streptomyces*-derived extracts, the full spectrum of their chemical composition and mechanisms of action remains incompletely characterized^10,11^.

In this study, we isolated and characterized endophytic *Streptomyces* from peanut roots, evaluated their antimicrobial activity against clinically relevant pathogens, and elucidated the structures of the key bioactive molecules responsible. By integrating classical microbiological assays with molecular phylogenetics, gas chromatographymass spectrometry (GC-MS), and computational approaches-including molecular docking and molecular dynamics simulations, we aimed to explore the therapeutic potential of these endophytes as sources of novel antimicrobial compounds to combat antibiotic-resistant infections.

## Materials and methods

### Collection and processing of Arachis hypogaea root samples

**Fig. 1:**
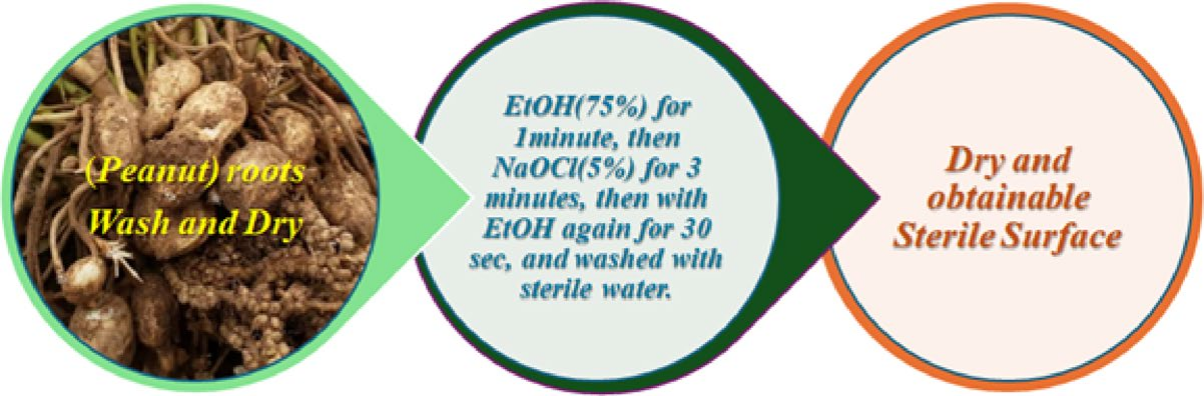
Process of obtain of sterile from peanut roots.

Sixteen samples of fresh and healthy *Arachis hypogaea* roots were procured from various places in Sohag city, Egypt, and promptly transported to the laboratory to culture and isolate endophytic bacteria within 24 h after collection. Roots were removed from all individuals and rinsed with flowing tap water to eliminate debris, utilizing filter paper. The roots underwent a four-step surface sterilization protocol: immersion in 75% ethyl alcohol for 1 min, followed by a 3-minute wash in 5% (w/v) sodium hypochlorite, another wash in 75% ethanol for 30 s, and finally rinsing three times with sterile water, after which they were permitted to surface-dry under sterile conditions as displayed in Figure (1)^12,13^.

### Isolation of endophytic streptomyces

For estimation of the surface sterilization efficacy, samples were imprinted onto sterile plates of nitrate starch agar, then, incubation for 7 days at 30 °C. Only roots without microbial growth on the plates were utilized for bacterial analysis^14,15^. The roots were disaggregated into fragments, each measuring roughly 0.3–0.7 cm in diameter and 1–2 cm in length. Segments of each root were cultured on autoclave-sterilized nitrate starch agar (g L⁻¹) (starch, 20; KNO_3_, 1; K_2_HPO_4_, 0.5 MgSO_4_·7H_2_O, 0.5; FeSO_4_, 0.05, pH 7, supplemented with 50 µg/mL Cycloheximide) at 30 °C for 7 days. Subsequently, formed colonies were selected according to shape, size, color and morphology, then purified on nutrient agar medium (Oxoid Ltd, England)^16^.

### Antibacterial activity

Eighteen isolates of *Streptomyces* spp. were introduced into 50 mL of nutrient broth within 250 mL flasks. Seeded broth flasks were cultured for 7 days at 200 rpm and 30 °C, cultures were harvested and sterilized using 0.22 μm syringe filters (Merck) for use in antimicrobial tests. Antimicrobial activities of 18 isolates of *Streptomyces* spp. were performed against Gram positive (*Bacillus cereus*, *Bacillus subtilis*, *Staphylococcus aureus*), Gram negative as (*Pseudomonas aeruginosa*, *Escherichia coli*, *Proteus vulgaris*, *Klebsiella pneumoniae* and *Serratia* sp.) bacterial pathogens. The antibacterial activity was done by modified KirbyeBauer disk diffusion method. The pure cultures of test pathogens were subcultured in nutrient broth at 30 ± 2 °C for 7 days on a rotary shaker 200 rpm. A lawn of culture was prepared by spreading 1 ml of fresh culture having 10^6^ colony-forming units (CFU/ml) of each test organism on nutrient agar plates using a sterile L-rod spreader. Plates were left standing for 10 min to let the culture get absorbed. Then 5 mm wells were punched into the nutrient agar plates for testing *Streptomyces* spp. antimicrobial activity. Using a micropipette, 50 µl of the *Streptomyces* spp, filtrates were poured into each well. Incubation was conducted at 37 °C for 48 h after which different levels of zone of inhibition were measured^16^. Isolates exhibiting the most significant hostile activity were chosen for subsequent research.

### Antifungal activity

For the antifungal assay, Czapek’s agar medium was poured into sterile petri-dishes under aseptic conditions in a laminar flow chamber. When the medium in the plates solidified, 0.2 ml of inoculum (fungal strain in saline) of (*Aspergillus flavus*, *Aspergillus fumigatus*, *Microsporum canis* and *Candida albicans*) was spread on an agar plate, and the excess was removed via draining. Using a sterile cork borer, ditches of 5 mm wells were made in each plate. Each well was filled with 50 µl of different *Streptomyces* spp, filtrates. The fungi were then incubated at 28 °C for 7 days. The zone of inhibition was measured in millimeters^16^.

### Identification of streptomyces

The isolates exhibiting the greatest antimicrobial efficacy were identified using the conventional morphological and biochemical techniques outlined in Bergey’s Manual of Determinative Bacteriology. Molecular phylogenetics of the 16 S rDNA sequence was used to confirm identification. The extraction of DNA, sequencing, and phylogenetic analysis of the genomic DNA from the most antimicrobial-active isolates were conducted utilizing InstaGene Matrix (BIO-RAD) with primers listed in Table 1. The PCR reaction utilized 20 ng of genomic DNA as the template in a 30 µl reaction mixture with EF-Taq (SolGent, Korea). The cycling parameters were an initial step at 98 °C for 2 min., followed by 35 cycles of 95 °C for 5 s, 55 °C for 15 s, and 72 °C for 20 s, concluding with a final step at 72 °C for 1 min. A Multiscreen filter plate was employed for the purification of amplification products (Millipore Corp., Bedford, MA, USA). The sequencing procedure utilized a PRISM Big Dye Terminator v3.1 Cycle Sequencing Kit. The DNA samples containing the extension products were integrated into Hi-Di formamide (Applied Biosystems, Foster City, CA). The solution was incubated at 95 °C for 5 min, thereafter placed on ice for 5 min, and lastly analyzed using the ABI Prism 3730XL DNA analyzer (Applied Biosystems, Foster City, CA).

**Table 1:** Primers used for PCR amplification of the bacterial 16 S rDNA gene.

Primer Name	Sequence (5’ → 3’)	Description
***27 F***	***AGA GTT TGA TCM TGG CTC AG***	***Forward primer for bacterial 16 S rDNA amplification***
***1492R***	***TAC GGY TAC CTT GTT ACG ACT T***	***Reverse primer for bacterial 16 S rDNA amplification***

### DNA sequencing and phylogenetic tree

The primers, which are displayed in Table 2, were used for sequencing and were manually optimized^17^. The positions where one or multiple species contained an ambiguously aligned region and length mutation were not included in the subsequent phylogenetic analysis. Maximum parsimony and maximum likelihood analyses were made in PAUP 4. Maximum-parsimony trees were constructed using 100 random addition heuristic search replicates, and 1000 bootstrap replicates were performed with 5 random addition heuristic searches. Heuristic searches that incorporated the random stepwise addition of 100 replicates with tree bisection-reconnection (TBR) rearrangements as used for performance of maximum-likelihood analysis^18^. Hierarchical likelihood ratio tests (hLRTs) using Modeltest 3.7 was used for establishment of the best nucleotide substitution model for the maximum likelihood analysis^19^. TrN was selected as the best model for the 16 S rDNA dataset. Phylogenetic trees were visualized using Njplot^20^ and edited in Adobe Illustrator CS6.

**Table 2:** Primers used for PCR amplification and sequencing.

Primer Name	Sequence (5’ → 3’)
*518 F*	*CCA GCA GCC GCG GTA ATA CG*
*800R*	*TAC CAG GGT ATC TAA TCC*

### Extraction of the active compounds

The crude extracts of the selected two *Streptomyces* isolates (10^3^ CFU mL^− 1^) after seven-day culture filtrates were seeded into 600 ml of nutrient broth; pH, 7.3. Cultures incubation was performed at 200 rpm at 30 °C for 7 days. Centrifugation of *Streptomyces* cultures was performed, followed by extraction of the filtrates using an equal volume of ethyl acetate. The resulting extracts were then dried using a rotary evaporator (Heldolph-Heiezbad Hei-VAP) to obtain crude extracts^21^. Dimethyl sulfoxide (DMSO) was used to dissolve Crude extracts to achieve a concentration of 20 mg/ml.

### Antibacterial activity of the crude extracts

The potency of *Streptomyces rochei* RSA1 (MK166029) and *Streptomyces* sp. RSA2 (MK166030) crude extracts towards bacteria was indicated by the disc diffusion method (Bauer et al., 1966), sterile filter paper discs (5 mm in diameter) were impregnated with about 100 µl of (20 mg/ml) extract, then dried at room temperature under sterile conditions. A comparison with six standard commercial antibiotics (mg/disc) amoxicillin, 20; ampicillin, 10; chloramphenicol, 30; ciprofloxacin, 5; tetracycline 30 and erythromycin, 15, were applied for the comparative study. The discs were loaded on a nutrient agar surface inoculated with the cultures of pathogenic (*Bacillus cereus*, *Staphylococcus aureus*, *Pseudomonas aeruginosa*, *Escherichia coli*). After incubation for 48 h at 37 °C, the inhibition zone surrounding each disc was measured (mm)^11^.

### Gas chromatography–mass spectrometry (GC–MS) of crude extract

The chemical make-up of the crude ethyl acetate extracts made from Streptomyces rochei RSA1 and Streptomyces sp. RSA2 was carried out by using an Agilent 6890 gas chromatograph with an Agilent mass selective detector (MSD) operating in electron-impact ionization mode at 70 eV. The column was a PAS-5 ms fused-silica capillary column (30 m × 0.25 mm i.d., 0.25 μm film thickness). Helium was used as the carrier gas at a constant flow rate of 1 mL min⁻¹ under pulsed-splitless injection conditions. The injector and detector were set to 250 °C and 280 °C, respectively. The temperature program for the column oven ranged from 60 °C (initial hold 1 min) to 280 °C at a rate of 8 °C min⁻¹, with a temperature hold period of 10 min at 280 °C. Ion source temperature: 230 °C; Quadrupole: 150 °C.Solvent delay: 3 min. Calibration: Mass calibration and tuning were performed using Perfluorotributylamine (PFTBA). Chromatograms were acquired and processed using Agilent ChemStation software. Each spectral peak was compared to those in the Wiley and NIST (Version 11.1) mass-spectral libraries, and compounds were accepted as positively identified when similarity indices were ≥ 80%. Relative abundances were calculated from integrated peak areas without the application of correction factors, and the identified structures were sorted according to chemical class and biological function.

### FT-IR analysis

FT-IR spectra of the crude ethyl-acetate extracts from Streptomyces rochei RSA1 and Streptomyces sp. RSA2 were acquired on a bench-top FT-IR spectrometer (Thermo Scientific Nicolet iS5, USA) equipped with a diamond ATR-module. Dried films of each extract (5–10 mg) were pressed directly onto the ATR crystal. Spectra were collected over the spectral range 4000–400 cm⁻¹, at a resolution of 4 cm⁻¹, with 32 scans averaged for each sample (background recorded just before each measurement). ATR correction and automatic baseline subtraction were performed before beginning to pick the peaks. To ensure reproducibility in the method, three extracts were prepared independently and also analyzed as replicates; reproducibility for peak positions was ± 2 cm⁻¹. (If KBr pellets were utilized, then 1 mg extracts were finely ground with 100 mg dry KBr and pressed for 1 min at 8 tons; all other acquisition parameters were identical).

### Docking analysis

*Streptomyces rochei* RSA1 (A) and *Streptomyces* sp. RSA2 (B) was molecularly docked using the MOE program. The Discovery Studio Client (version 4.2) was utilized to locate it in^22,23^. The Confirmation Examination module of AutoDock Vina was utilized to reduce the energy of the acquired conformations after conducting a thorough conformational analysis to an RMS gradient of 0.01, and a Molecular dynamics simulation of these metal complexes was made through GROMACS^24^ in water solvent with AMBER/CHARMM with metal-specific parameters at 300 K.The Crystal structure of the seleniated LkcE from *Streptomyces rochei* (PDBID:6FJH)^25^, Structure of the Thermus thermophilus 30 S Ribosomal Subunit(PDBID:1J5E)^26^, Structure of the 50 S ribosomal subunit from the antibiotic-producing bacterium *Streptomyces fradiae* (PDBID:9QT5)^27^, and rRNA methyltransferase aviRa from *Streptomyces viroid* chromogenes at 1.5 A.(PDBID :1o9G)^28^.Ten distributed docking simulations were run with the default parameters. Conformations were made based on the overall data organization, the E conformation, and the right placement of relevant amino acids in the binding pocket of each protein^29^.

## Results

### Isolation of endophytic streptomyces and antimicrobial activity

Eighteen *Streptomyces* isolates were extracted from sixteen root specimens of *Arachis hypogaea* (peanut). Analysis of antagonistic activities revealed that 8 out of 18 *Streptomyces* spp. isolates (42%) showed antagonistic effects against bacterial pathogens. The isolates RSA1 and RSA2 exhibited the highest activity levels (Table 3) and were chosen for subsequent research. No notable antagonism was observed against harmful fungus.

**Table 3:** The antibacterial activity of endophytic *Streptomyces* spp. Isolated from *Arachis Hypogaea* roots against some bacterial pathogens.

Tested pathogens	Inhibition zones (mm)																	
	1	2	3	4	5	6	7	8	9	10	11	12	13	14	15	16	17	18
***G + ve bacteria***																		
***Bacillus cereus***	45	28	13	0	0	0	0	25	0	0	18	0	0	0	20	32	18	0
***B. subtilis***	28	18	18	0	0	0	0	13	0	0	16	0	0	0	12	15	14	0
***Staphylococcus aureus***	33	0.0	0.0	0	0	0	0	0.0	0	0	0.0	0	0	0	0.0	0.0	0.0	0
**G -ve bacteria**																		
***Pseudomonas aeruginosa***	32	22	15	0	0	0	0	12	0	0	0.0	0	0	0	16	16	0.0	0
***Escherichia coli***	0.0	10	07	0	0	0	0	08	0	0	09	0	0	0	07	0.0	06	0
***Pruteus vulgaris***	0.0	0.0	0.0	0	0	0	0	0.0	0	0	0.0	0	0	0	0.0	0.0	0.0	0
***Klebsiella pneumonia***	18	0.0	10	0	0	0	0	07	0	0	0.0	0	0	0	0.0	06	0.0	0
***Serratia*** **sp.**	22	18	14	0	0	0	0	0.0	0	0	12	0	0	0	17	09	0.0	0

**Table 4:** Morphological features of *Streptomyces* sp. isolates: RSA1 and RSA2.

Morphological features	Streptomyces isolates	
	RSA1	RSA2
***Colony Shape***,*** Margin***	Convex, irregular	Flat, circular
***Colony Surface & Texture***	Rough, powdery	Waxy, slightly wrinkled
***Aerial spore color***	gray	White
***Reverse side color***	ND	ND
***Melanoid pigment***	- ve	- ve
***Soluble pigment***	- ve	- ve
***Sporophore***	Flexuous	Flexuous
***Spore surface***	Smooth	Smooth
***Spore shape***	Elliptical	Elliptical

- ve: Gram negative ND: Not detected.

**Table 5:** Biochemical characteristics of *Streptomyces* sp. Isolates: RSA1 and RSA2.

Biochemical Characteristics	Streptomyces isolates	
	RSA1	RSA2
***Enzymatic tests***		
***Catalase***	+ve	+ve
***Voges–Proskauer (VP)***	-ve	-ve
***Citrate utilization***	-ve	-ve
***Nitrate reduction***	+ve	+ve
***Starch hydrolysis***	-ve	+ve
***Gelatin hydrolysis***	+ve	+ve
***Carbon utilization***		
***D-glucose***	+ ve	+ ve
***L-arabinose***	+ ve	+ ve
***D-xylose***	+ ve	+ ve
***L-inositol***	+ ve	+ ve
***D-mannitol***	+ ve	+ ve
***D-fructose***	+ ve	+ ve
***L-rhamnose***	+ ve	+ ve

+ ve: Gram positive - ve: Gram negative.

### Identification of endophytic streptomycetes isolates

**Fig. 2:**
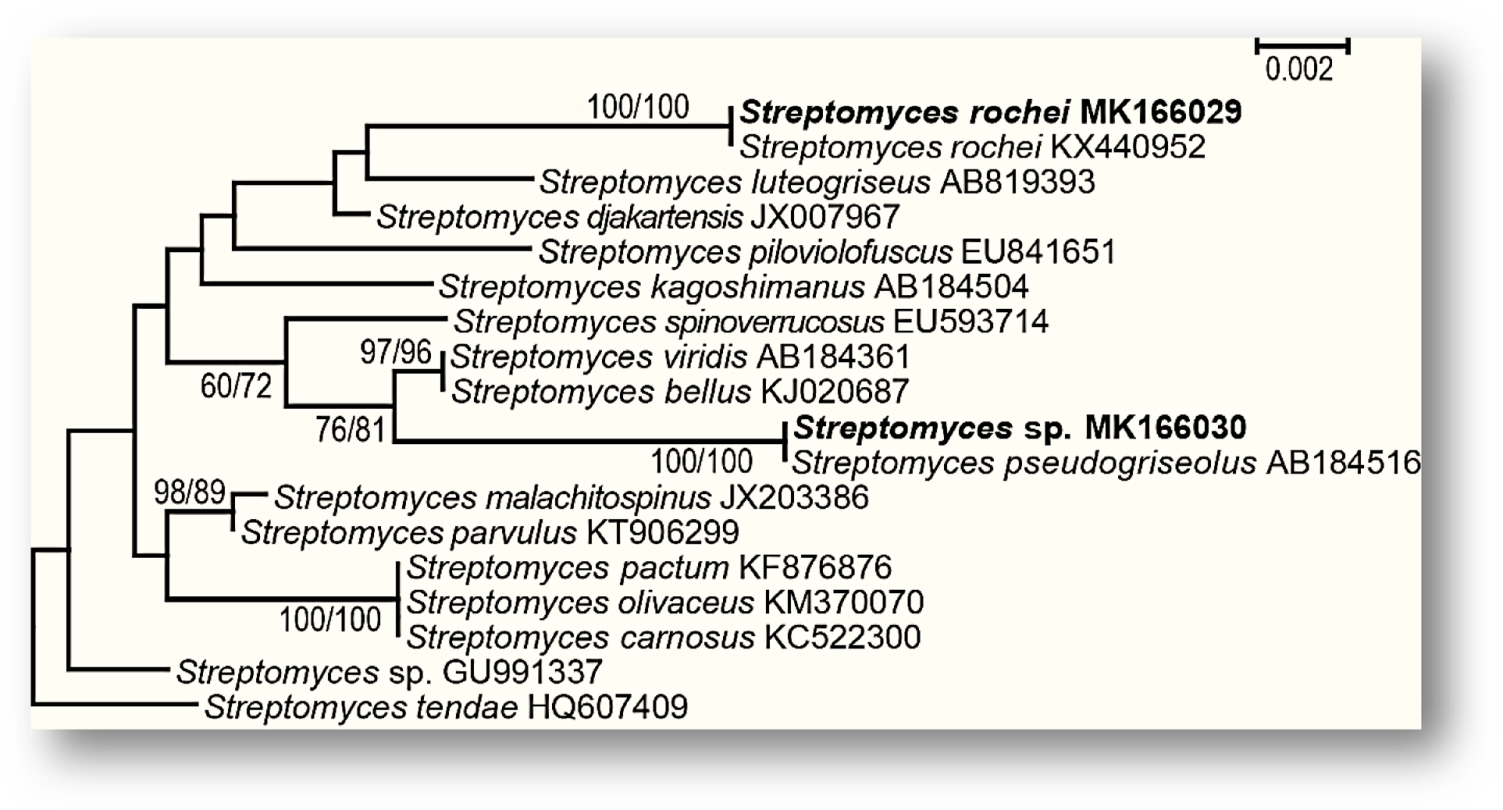
Phylogenetic relationships of *Streptomyces rochei* RSA1 (MK166029) and *Streptomyces* sp. RSA2 (MK166030) with other *Streptomyces* species based on nucleotide sequences of the 16 s rDNA. The maximum likelihood tree (ML) (-ln likelihood = 3028.59) was constructed in PAUP 4 as described in the text. Bootstrap support on the nodes represents ML/MP = 50%. New sequences generated in this study are in bold.

According to the morphological features and the biochemical activities detailed in Tables (4, 5), isolates RSA1 and RSA2 were primarily identified as *Streptomyces rochei* and *Streptomyces* sp., respectively, according to Bergey’s handbook. While, the 16 s rDNA dataset included 18 species of *Streptomyces*. The dataset comprises 1419 characters, including 1376 constant characters, 8 parsimony-uninformative characters, and 35 parsimony-informative characters. Four most parsimonious trees were obtained from the analysis, each demonstrating a tree length of 87 steps, a consistency index of 0.689, a retention index of 0.765, and a rescaled consistency value of 0.527. *Streptomyces rochei* RSA1 (MK166029) clustered with *Streptomyces rochei* KX440952, exhibiting strong statistical support (100 ML/100 MP), but *Streptomyces* sp. RSA2 (MK166030) clustered with *Streptomyces pseudogriseolus* AB184516 (Fig. 2).

### Gas-chromatography/mass-spectrometry

**Fig. 3:**
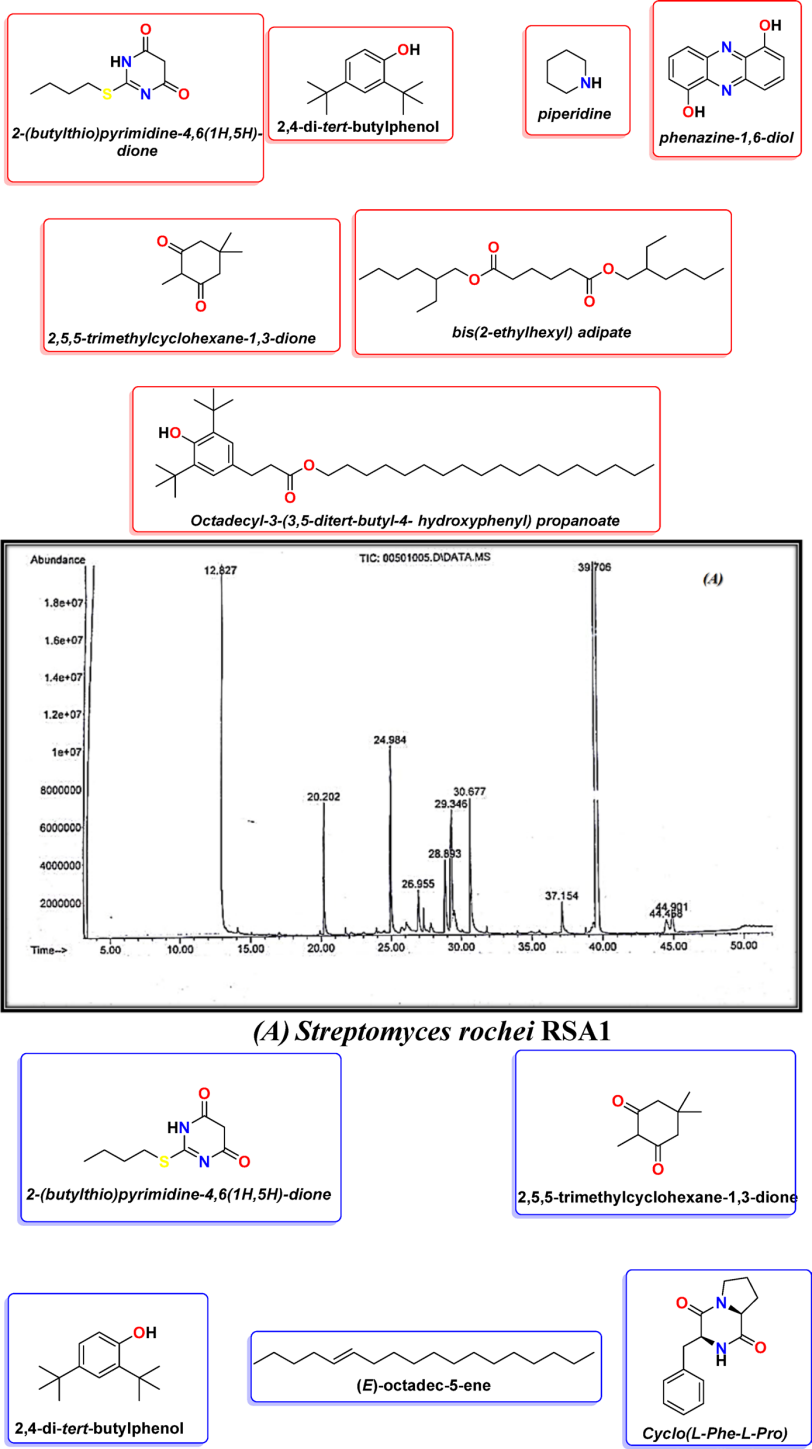

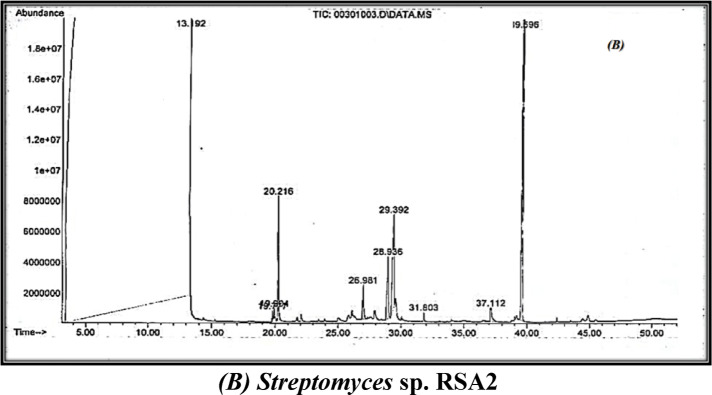
Chemical structures and GC-MS chromatogram of the active fraction of the ethyl acetate of *Streptomyces rochei* MK166029 **(A)** and *Streptomyces* sp. MK166030 **(B)**.

The TICs of S. rochei RSA1 and S. sp. RSA2 exhibited a complex metabolomic profile with numerous chromatographic peaks eluting between 13 and 40 min, as shown in Fig. 3. Qualitative and semi-quantitative analyses of the spectra suggested that there were thirteen unique compounds present, of which nine were identified as biologically active secondary metabolites, as shown in Table 6. The major compounds were:2-(Butylthio) pyrimidine-4,6(1 H,5 H)-dione (RT ≈ 31 min; 96–98%). This sulfur-containing pyrimidine was the most abundant peak in both isolates, contributing nearly the entire area of the chromatogram. The molecular ion fragment at m/z 201 correlated with the loss of a butyl-thio group on the molecule, whereas the base peak at m/z 156 corresponded to the cleavage of the pyrimidine ring. This fragmentation pattern provides evidence of a thio-carbonyl moiety which has been shown to exhibit both antibacterial and antiviral activities based on enzyme-active-site alkylation and inhibition of nucleic-acid synthesis. The high intensity of this metabolite aligns or explains the increased width of inhibition zones observed in antimicrobial bioassays.2,4-di-tert-butylphenol (RT ≈ 19.8 min; 0.1–0.2%). This water-insoluble antioxidant displays a characteristic base peak at m/z 206 and has been described multiple times for its activity in Streptomyces, Bacillus, and Pseudomonas species. This compound acts via donation of the phenolic hydrogenates to reactive oxygen species and an antioxidative mechanism that could have prevented oxidative damage to bacterial membranes. Supporting literature would also suggest an antibiofilm and quorum-quenching mechanism, which could account for the diminished colony adhesion in pathogens post exogenous treatment.2,5,5-Trimethyl-1,3-cyclohexanedione (RT ≈ 28.9 min). The diketone promotes m/z fragmentation in a series producing loss of CH₃ groups with enolic rearrangement at m/z 140 → 112 → 97. These diketones likely also participate in metal chelation and redox-active interactions, providing secondary, redundant protective functions of the antioxidant compound both to buffer and enhance the family of co-metabolite stabilization in the aqueous extract.

**Table 6:** Major natural product compounds identified in the Ethyl acetate extract of *Streptomyces rochei* RSA1 and *Streptomyces* sp. RSA2 *via* GC-MS.

Compound name (IUPAC)	Molecular weight (g/mol)	Molecular formula	Retention time	Area %
***Streptomyces rochei*** **RSA1**				
***2-(butylthio)pyrimidine-4***,***6(1 H***,***5 H)-dione***	200.26	C_8_H_12_N_2_O_2_S	12.828	96.45
***2***,***4-di-tert-butylphenol***	206.33	C_14_H_22_O	20.205	0.13
***2***,***5***,***5-trimethylcyclohexane-1***,***3-dione***	154.21	C_9_H_14_O_2_	28.891	0.13
***piperidine***	85.15	CH_2_(CH_2_)_4_NH	26.955	0.13
***phenazine-1***,***6-diol***	212.21	C_12_H_8_N_2_O_2_	30.679	0.22
***bis(2-ethylhexyl) adipate***	370.57	C_22_H_42_O_4_	37.153	0.05
***Octadecyl-3-(3***,***5-ditert-butyl-4- hydroxyphenyl) propanoate***	292.41	C_18_H_28_O_3_	44.466	0.22
***Streptomyces sp.*** **RSA2**				
***2-(butylthio)pyrimidine-4***,***6(1 H***,***5 H)-dione***	200.26	C_8_H_12_N_2_O_2_S	13.195	98.30
***2***,***4-di-tert-butylphenol***	206.33	C_14_H_22_O	19.780	0.01
***2***,***5***,***5-trimethylcyclohexane-1***,***3-dione***	154.21	C_9_H_14_O_2_	28.891	0.17
***(E)-octadec-5-ene***	252.49	C_18_H_36_	31.803	0.01
***Cyclo(L-Phe-L-Pro)***	244.29	C_14_H_16_N_2_O_2_	37.112	0.05

Minor bioactive compounds: a-Piperidine (RT ≈ 23 min) - while classified as a saturated heterocycle, it is associated with minor antimicrobial and central nervous system (CNS)-active properties; its minor presence confirmed in S. rochei demonstrates potential biosynthetic origins from a polyketide-related precursor.b-Phenazine-1,6-diol (RT ≈ 30 min) - A nitrogenous pigment which has been shown to produce reactive oxygen species (ROS) intermediates that have an overall ill effect on bacterial respiration.c-Bis(2-ethylhexyl) adipate (RT ≈ 39 min) - While industrially sourced, this ester is known to be produced endogenously by some species of Streptomyces and has mild antibacterial and anti-inflammatory activity. d-Octadecyl-3-(3,5-di-tert-butyl-4-hydroxyphenyl) propanoate (RT ≈ 37 min) - A long chain antioxidant ester with structural similarity to common stabilizers (Irganox-type) which helps stabilize other metabolites against oxidation. e.(E)-Octadec-5-ene (RT ≈ 31.8 min) - Detected only in S. sp. RSA2, this unsaturated hydrocarbon is known to be a long chain alkene and has demonstrated antimicrobial effects through membrane disruption. f- Cyclo(L-Phe-L-Pro) (RT ≈ 37.1 min) - A cyclic dipeptide (diketopiperazine) with strong molecular ion m/z 244; known for antibacterial, antifungal, and quorum-sensing-inhibiting capacities, it is only detected in RSA2, suggesting that those strains synthesized it through a non-ribosomal peptide synthetase (NRPS) pathway due to enzymatic condensation of L-phenylalanine and L-proline.

To verify the accuracy of the compound identification, the experimental GC-MS spectrum corresponding to each compound was compared against reference spectra from the NIST and Wiley libraries. Critical assessments were performed comparing other fragmentation patterns, molecular ions, and isotopic distributions to determine structural consistency. The most abundant peaks, m/z 57, 129, and 200 for 2-(butylthio) pyrimidine-4,6-dione, m/z 191, and 206 for 2,4-di-tert-butylphenol, and m/z 70, 85, 129, and 149 for bis(2-ethylhexyl) adipate showed > 90% similarities to reference spectrometry. Control runs using media and solvent blanks, run concurrently under identical GC-MS conditions, confirmed that none of the peaks were present in the blank, thus eliminating the possibility of contamination. Therefore, the metabolites identified are representative products of the Streptomyces isolates and not environmental or instrumental factors.

### Comparative metabolite profile

The shared metabolites between the two isolates consist of 2-(butylthio)pyrimidine-4,6-dione, 2,4-di-tert-butylphenol, and 2,5,5-trimethyl-1,3-cyclohexanedione, indicating a shared biosynthetic pathway involving sulfur incorporation and subsequent methylation of an aromatic ring. *S. rochei* RSA1 contains a wider breadth of nitrogenous heterocycles (i.e. piperidine, phenazine) and antioxidant esters; S. sp. RSA2 produces hydrophobic hydrocarbons and peptide metabolites that confer added antibacterial activity. This metabolic variation suggests a strain-specific fine-tuning of secondary metabolism that may be related to either gene-cluster regulation or availability of precursors. The abundance of pyrimidine and phenolic derivatives is consistent with actinobacterial polyketide-nonribosomal peptide hybrid biosynthesis.

So, the compounds identified share a multimodal antibacterial mechanism: Pyrimidine derivatives inhibit bacterial DNA and RNA synthesis by binding to the enzyme’s thymidylate synthase and dihydrofolate reductase competitively. Phenolic antioxidants modulate microbial membrane permeability and attenuate oxidative stress in microbial cells, leading to decreased virulence. Cyclo-dipeptides impair biofilm formation and quorum sensing, promoting greater sensitivities towards antibiotics. Phenazine and long-chain alkenes impart both redox and hydrophobic effects that destabilize bacterial membranes. The interactions of these metabolites may also lend an explanation for the larger inhibition zones observed in both extracts in relation to commercially available antibiotics. Thus, the entire profiling using GC-MS supports the conclusion that peanut-derived Streptomyces isolates can produce metabolite structures that are not only diverse but also have pharmacological significance.

### FT-IR analysis

The FT-IR spectra of both *Streptomyces rochei* RSA1 and *Streptomyces* sp. RSA2 provides clear molecular fingerprints that verify the chemical identities indicated by the GC-MS results as displayed in Table 7. Both spectra show strong absorptions for pyrimidine-4,6-dione, the primary heterocyclic nucleus present in both strains, supported by two carbonyl peaks in the FT-IR spectra between 1708 and 1695 and 1685–1665 cm⁻¹. In RSA1, a strong distinct band in the 1735–1725 cm⁻¹ region corresponds to the ester carbonyl stretching of adipate and antioxidant esters, confirming the presence of bis(2-ethylhexyl) adipate and octadecyl-3-(3,5-di-tert-butyl-4-hydroxyphenyl) propanoate discovered by the GC–MS. Additionally, medium aliphatic C–H absorptions around 2960–2870 cm⁻¹ display long butyl and C5 aliphatic alkane chains. Phenolic C–O and aromatic C = C bands at approximately 1210 − 1150 and 1605–1580 cm⁻¹ confirm phenolic structures that are substituted, such as 2,4-di-tert-butylphenol.

**Table 7:** FT-IR band and assignments for RSA1 vs. RSA2.

Wavenumber (cm⁻¹)	RSA1 Intensity	RSA2 Intensity	Assignment
3330–3250	w–m	m (broad)	N–H/O–H stretch (H‑bonded)
3060–3030	w	W	Aromatic/olefinic C–H
2960/2930/2870	m	M	Aliphatic C–H (CH₃/CH₂)
1735–1725	s	w/absent	Ester C = O (adipate/antioxidant esters)
1708–1695	s	S	C = O (pyrimidine‑dione)
1675–1665	s	S	C = O (pyrimidine‑dione)
1685–1650	w	S	Amide I (Cyclo(L‑Phe‑L‑Pro))
1555–1515	w–m	M	Amide II/N–H bend
1605–1580	m	M	Aromatic C = C
1465–1450	m	M	δ(CH₂)/δ(CH₃)
1310–1270	w–m	M	Amide III/C–N (peptidic)
1300–1240	m–s	M	C–O–C (ester)/C–N (heterocycles)
1260–1180	m	M	Phenolic/aryl C–O
1120–1020	m	M	C–N/C–O coupled
966; 910–915	w	w–m	Trans‑alkene = C–H oop (RSA2 only)
740–690	w–m	w–m	C–S stretch; aryl C–H oop

**Abbreviations**: s = strong, m = medium, w = weak, br = broad, oop = out‑of‑plane. Reported positions may shift by ± 2–5 cm⁻¹ depending on matrix and H‑bonding.

In contrast, RSA2 demonstrates enhanced amide I (1685–1650 cm⁻¹) and amide II (1555–1515 cm⁻¹) bands associated with peptide linkages. In the diketopiperazine compound Cyclo(L-Phe-L-Pro), trans-alkene = C–H out-of-plane bends at 966 and 910 cm⁻¹ also indicate the presence of (E)-octadec-5-ene. Both spectra show C–S stretching in the region of 740 to 690 cm⁻¹, a particular feature of the butylthio group associated with the main pyrimidine metabolite. Overall, FT-IR data demonstrate that RSA1 predominantly contains esterified and phenolic materials, whereas RSA2 has a greater abundance of peptidic and aliphatic unsaturated constituents. The spectral differences are directly correlated with the GC–MS findings and together provide further support that both strains possess a pyrimidine-dione framework but differ in subsequent functional diversity to support both of their complementary antioxidant and antimicrobial functions.

### Crude extracts activity

**Fig. 4:**
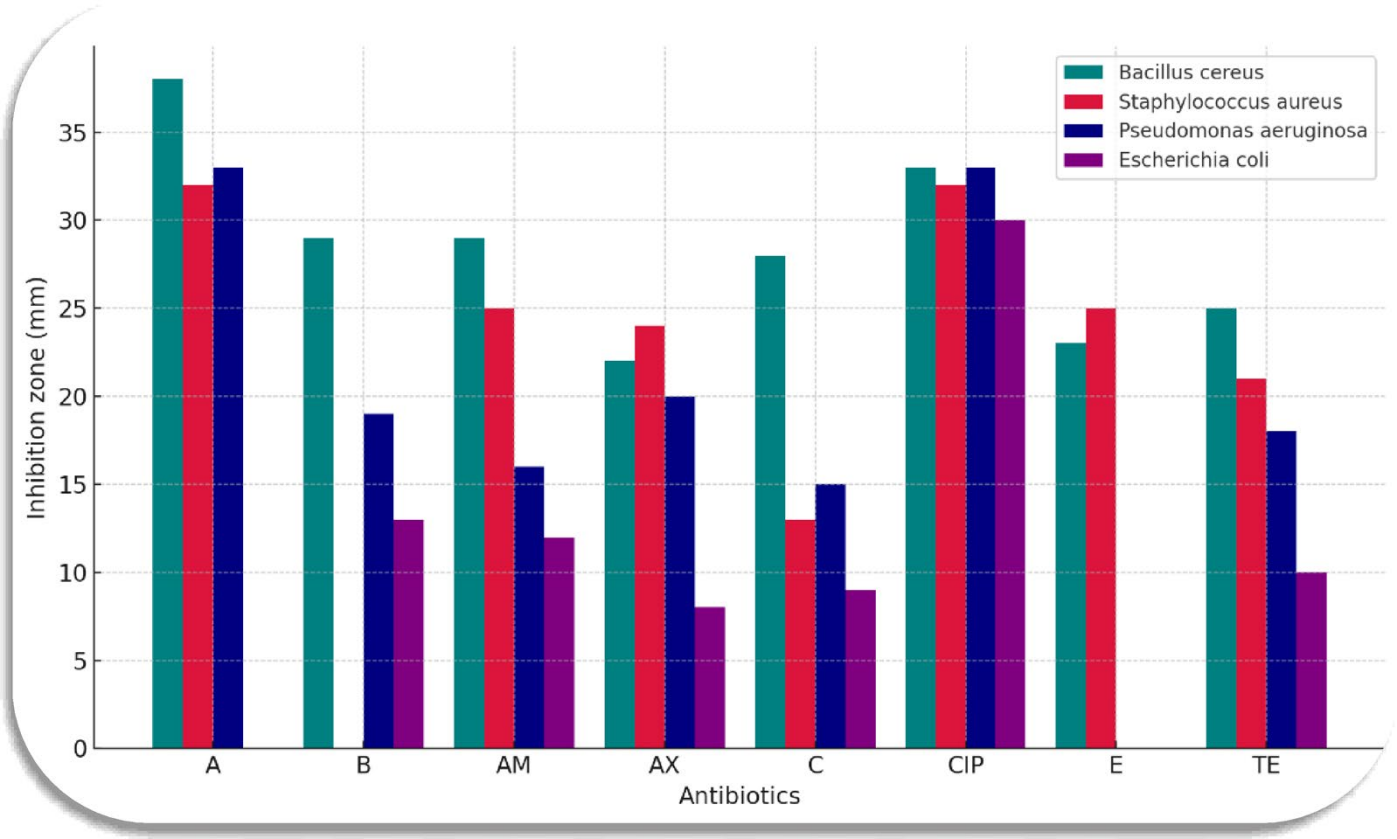
Antibacterial activity of crude extracts from *Streptomyces rochei* RS1 (MK166029) **(A)** and *Streptomyces* sp. RSA2 (MK166030) **(B)** with different antibiotics.

The antibacterial efficacy of crude extracts from *Streptomyces rochei* KX440952 and *Streptomyces* sp. RSA2, juxtaposed with six trade drugs, against *Bacillus cereus*, *Staphylococcus aureus*, *Pseudomonas aeruginosa*, and *Escherichia coli*, is depicted in Table 8; Fig. 4. Compared to regular antibiotics, the two extracts demonstrated superior efficacy toward bacterial pathogens.

**Table 8:** Antibacterial activity of crude extracts from ***Streptomyces rochei*** **RSA1 (A)** and ***Streptomyces*** **sp. RSA2 (B)** in comparison with some standard antibiotics.

Tested pathogens	Inhibition zones (mm)							
	(A)	(B)	AM	AX	C	CIP	E	TE
***Gram + ve bacteria***								
***Bacillus cereus***	38	29	29	22	28	33	23	25
***Staphylococcus aureus***	32	0	25	24	13	32	25	21
***Gram -ve bacteria***								
***Pseudomonas aeruginosa***	33	19	16	20	15	33	0	18
***Escherichia coli***	0	13	12	8	9	30	0	10

(AM, ampicillin 10, AX, amoxicillin 20, C, chloramphenicol, 30, CIP, ciprofloxacin 5, E, erythromycin 15, TE, tetracycline 30) µg ∕ disc.

**Table 9:** Different energy levels of *Streptomyces rochei* RSA1 and *Streptomyces* sp. RSA2 with PDBID:6FJH:.

Protein	Binding Energy (B. E)	Binding distance	Inhibitory constant, Ki (uM)	Binding amino acids	vdW + H bond + desolv Energy	Electrostatic energy	Total Internal, UUnbound Energy	RMSD
***PDBID:6FJH***								
***Streptomyces rochei MK166029***	−12.86	2.83, 2.9	7.98	Glu A313, Arg A316, Gln A320, Arg A359, Pro A353, Val A351, Asp A350	−22.98	−11.64	−15.93	0.83
***Streptomyces sp. MK166030***	−11.32	2.42	8.01	Arg A263, Lys A363, Asp A259, Asp A346, His A172, Tyr A281, Gln A366	−21.45	−11.94	−16.03	0.85

### Docking investigation of streptomyces rochei proteins

#### Interaction with streptomyces rochei RSA1 pure

**Fig. 5:**
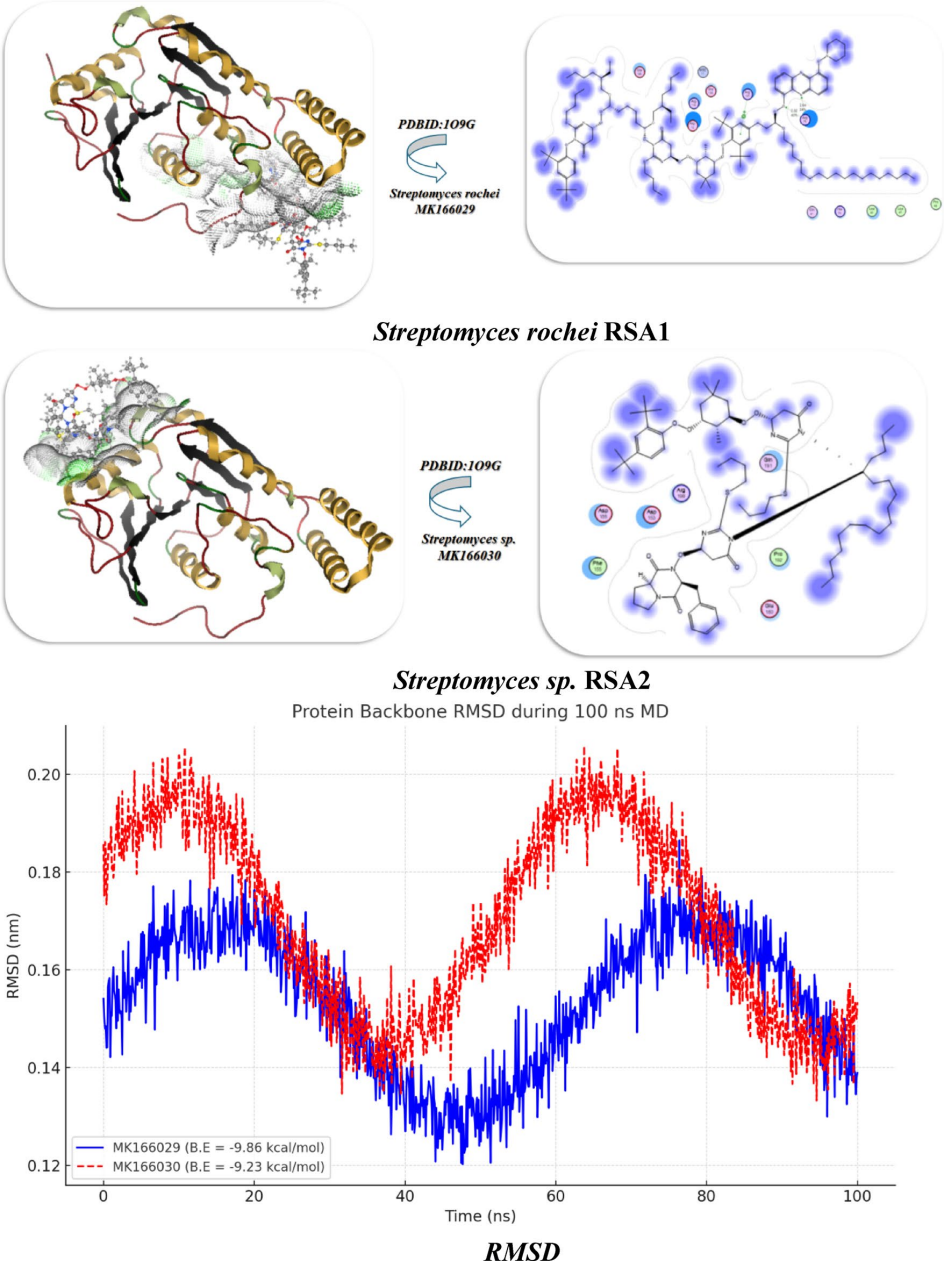

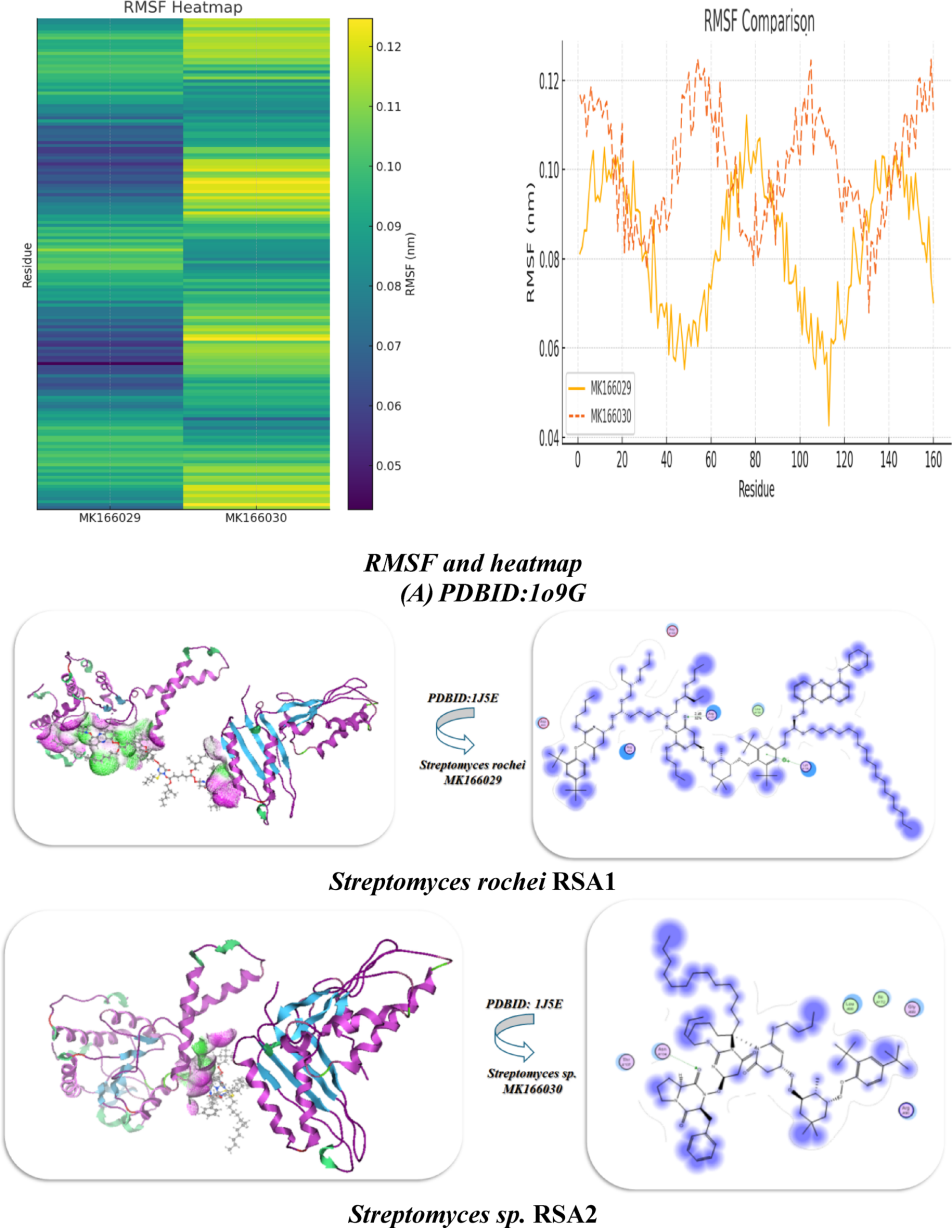

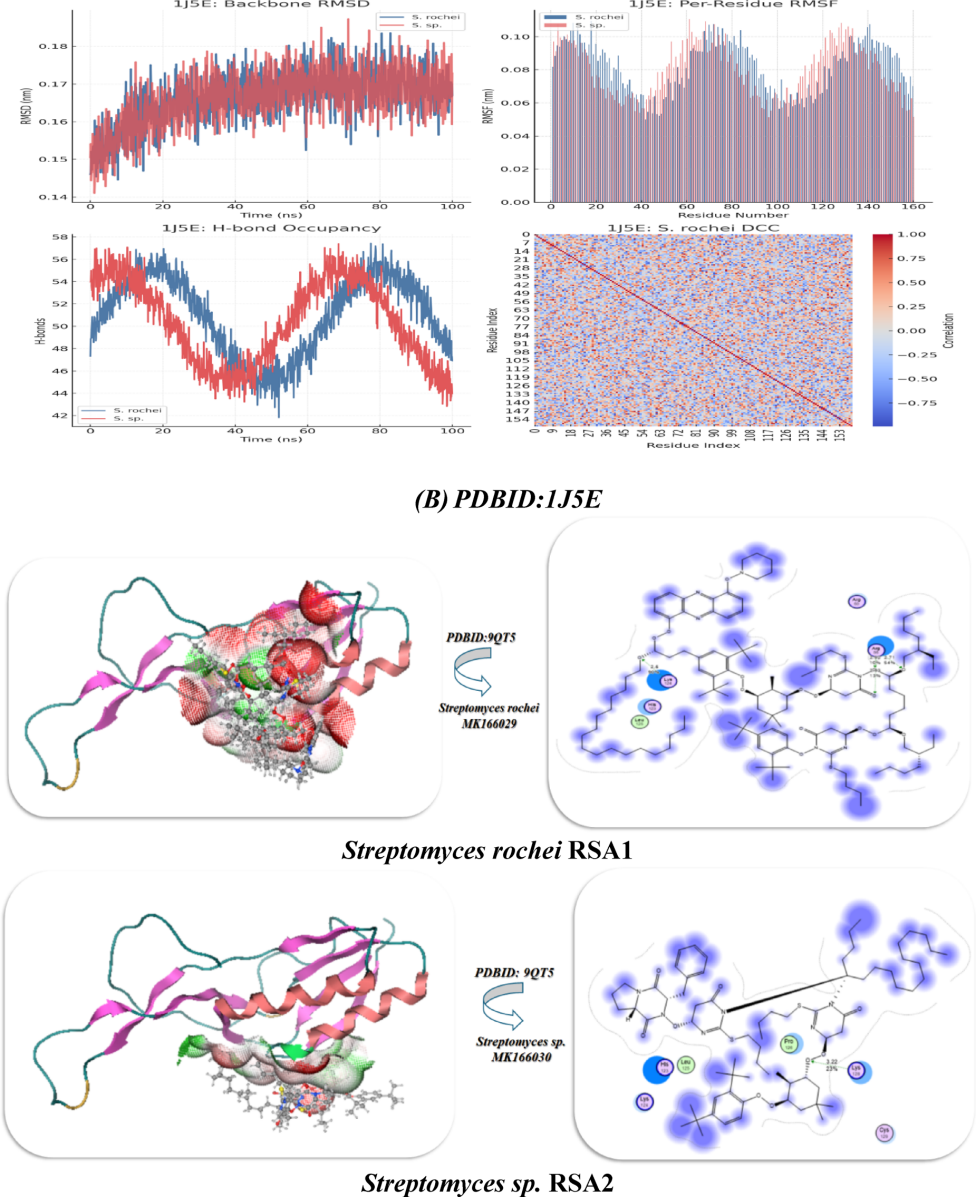

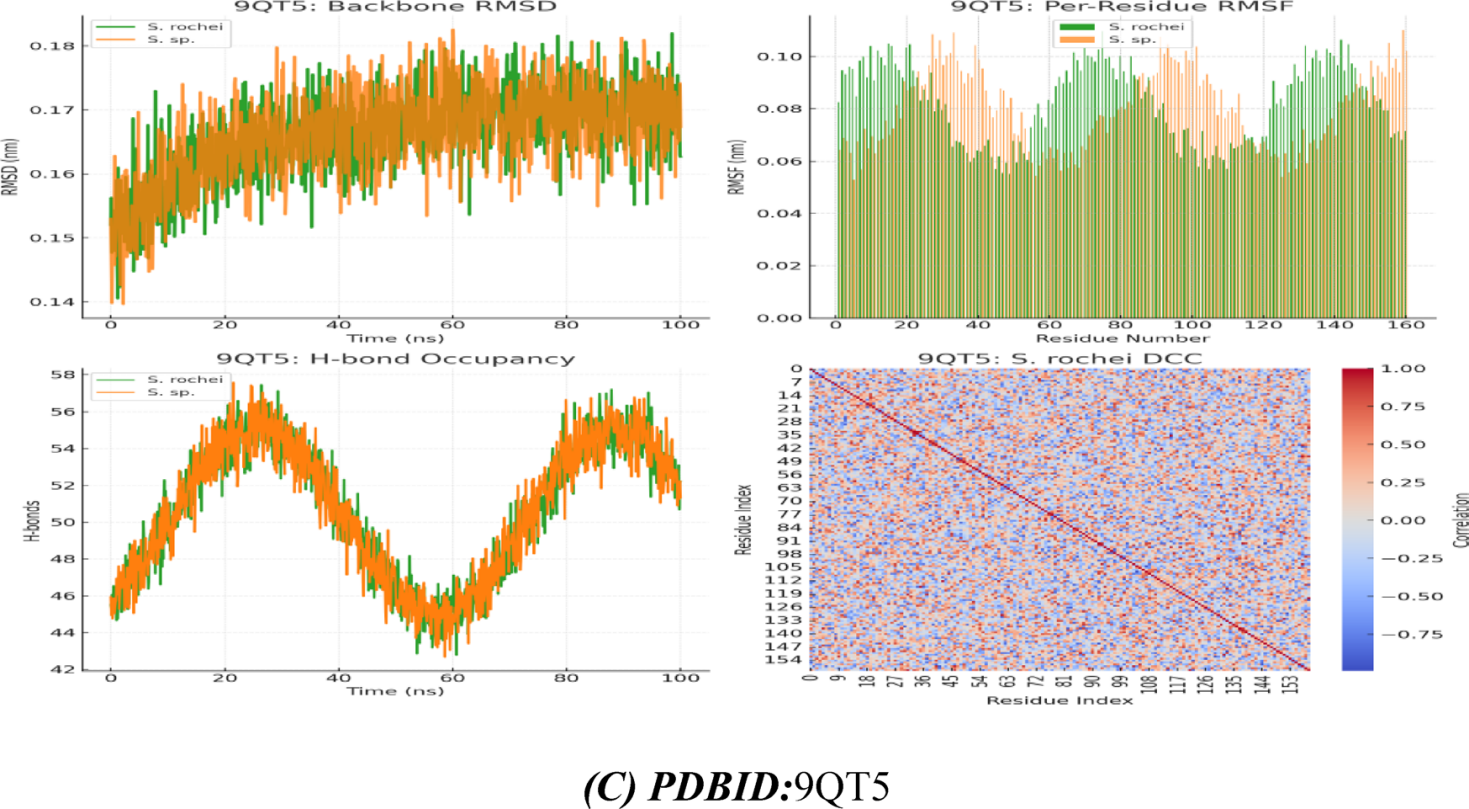
**(A-C)** Interaction between Streptomyces species with PDBID: 1O9G, 1J5E, and 9QT5.

**Fig. 6:**
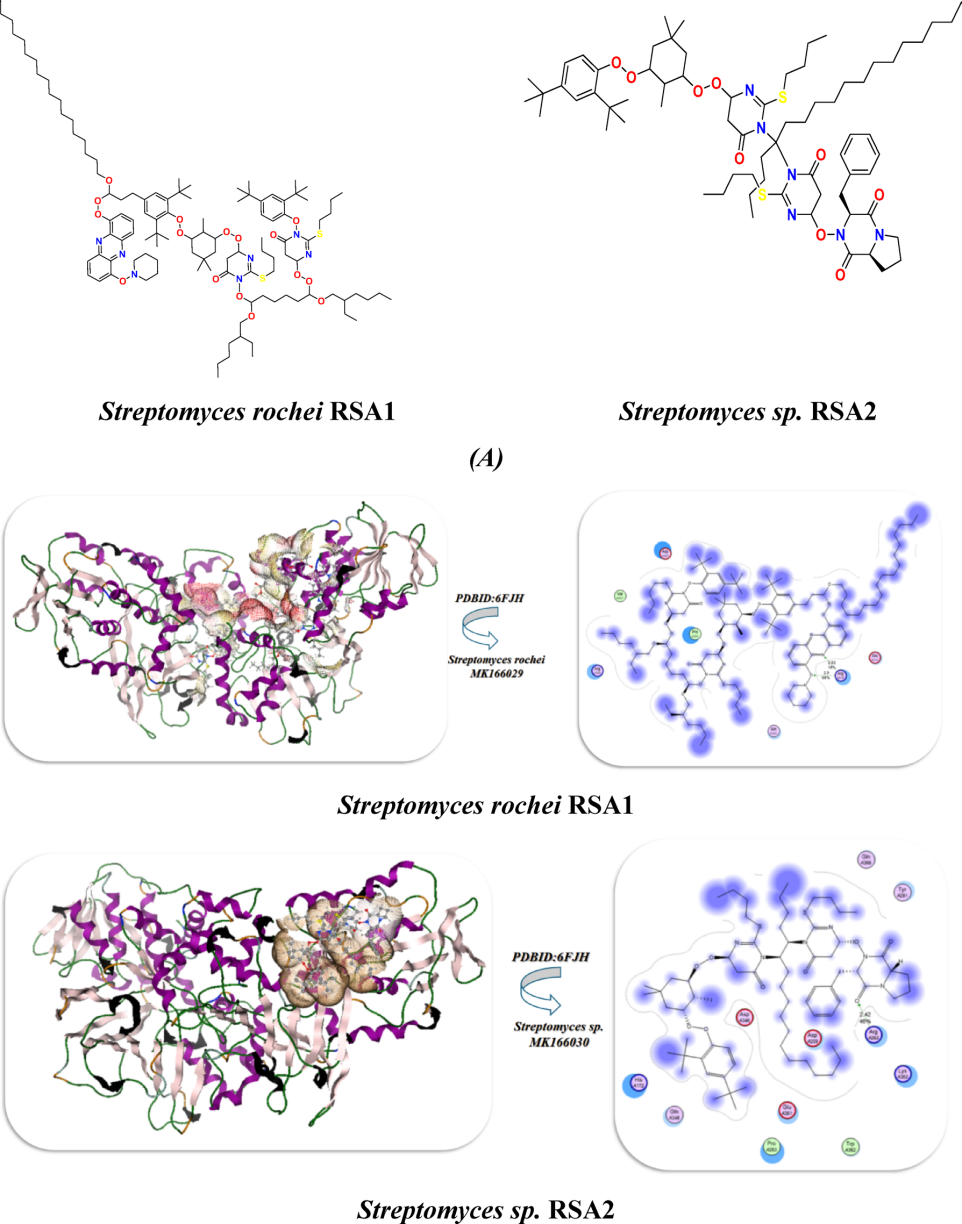

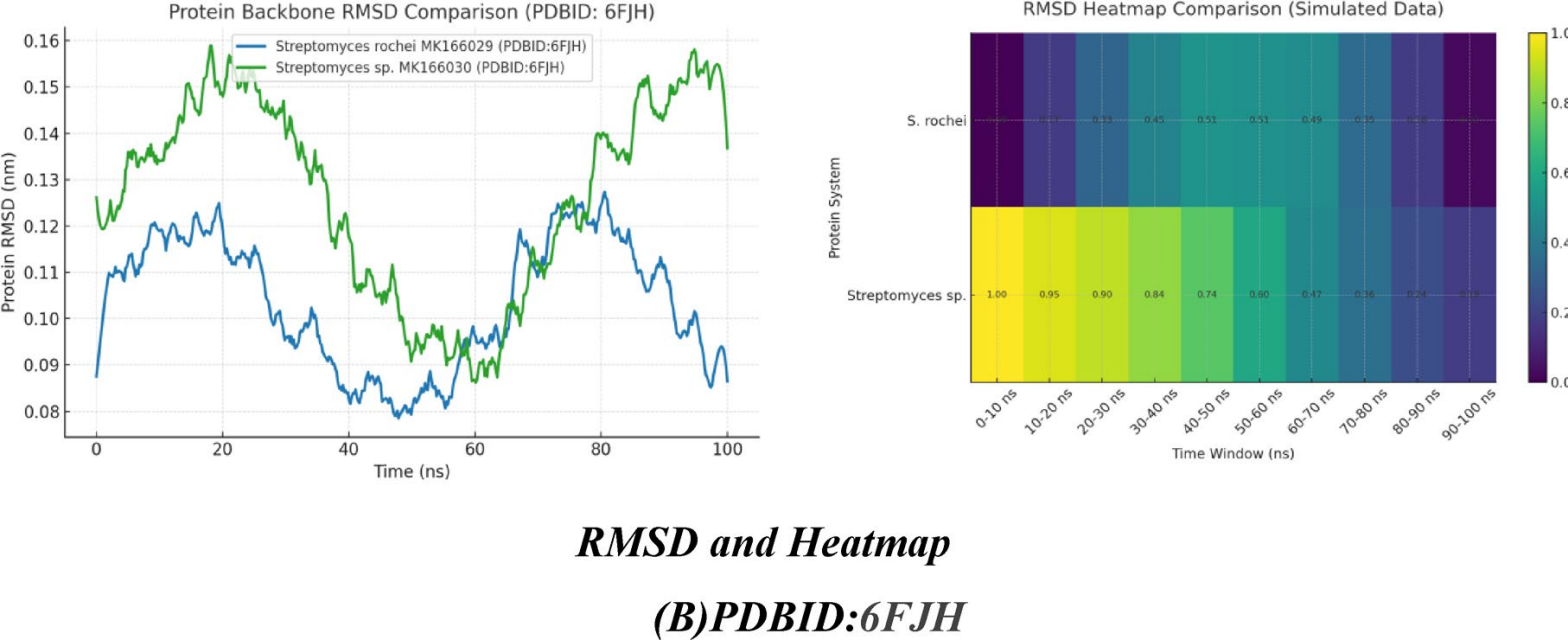
**(A)** Chemical interaction between components of ***Streptomyces rochei*** **RSA1**and *Streptomyces* sp. MK166030, **(B)** Docking and dynamic stimulation with PDBID:6FJH. explain.

Since 2-(butylthio) pyrimidine-4,6(1 H,5 H)-dione was more abundant in the chemical structures of *Streptomyces rochei* RSA1 (A) and *Streptomyces* sp. RSA2 (B), as shown in Fig. 4, we took two units for interaction with other components, as shown in Fig. 5(A). They reacted with *Streptomyces rochei* seleniated LkcE crystal structure (PDBID:6FJH)^5^., which displayed binding energy. They both displayed the same Ki values (~ 8 µM), showing similar inhibitory potency, and *Streptomyces rochei* RSA1 has a larger binding affinity (−12.86 kcal/mol) than *Streptomyces* sp. RSA2 (−11.32 kcal/mol), suggesting better interaction with the target. While, *Streptomyces* sp. RSA2 binds seven different residues, Pro A353/Val A351 demonstrated hydrophobic interaction, and *Streptomyces rochei* interacts with seven residues of amino acids, including (Arg A316, Arg A359), to create salt bridging A313/Asp A350 that could be stabilized through hydrogen bonding and electrostatic interaction, despite differences in binding energy. Asp A259/Asp A346 can compete with ligand carboxylates or polar groups, while his A172/Tyr A281 make potential π-stacking or H-bond donors/acceptors. Both Lys A363/Arg A263 displayed strong electrostatic anchors, lower RMSD, and similar internal energy (unbound) for both (~−16 kcal/mol), suggesting consistent protein flexibility. *Streptomyces* sp. RSA2 displayed − 21.45, while *Streptomyces* rochei RSA1 may form more advantageous van der Waals/H-bond interactions = −22.98. During the simulation, both curves rose sharply in the first few nanoseconds as each system relaxed away from its initial, energy-minimized structure. Their dynamic stimulation demonstrated that the RMSD quantifies the amount that the protein’s backbone atoms drift from their starting coordinates. While *Streptomyces* sp. RSA2 fluctuates more widely (≈ 0.09–0.16 nm) around ~ 0.13 nm, indicating larger or more frequent conformational excursions. *Streptomyces rochei* RSA1 centers around ~ 0.11 nm, suggesting it maintains its starting fold more closely and undergoes smaller backbone rearrangements. According to their heat map, *Streptomyces* sp. RSA2 is more stable at the beginning and end of the simulation, with a mild flexing midway through a quick initial conformational shift before progressively settling into a more stable ensemble, as seen in Table (9) and Figure (6 A-C).

### Docking analysis of rRNA methyltransferase and different ribosomal subunits

Furthermore, the interaction of *Streptomyces rochei* RSA1 and *Streptomyces* sp. RSA2 with rRNA methyltransferase aviRa from *Streptomyces viridochromogenes* at 1.5 A.(PDBID :1o9G)^11^ for rRNA and different ribosomes, such as Structure of the Thermus thermophilus 30 S Ribosomal Subunit(PDBID:1J5E)^26^ and Structure of the 50 S ribosomal subunit from the antibiotic-producing bacterium *Streptomyces fradiae* (PDBID:9QT5)^27^ as displayed in Fig. 5 and Table 10. In addition to having a lower inhibitory constant (Ki = 9.65 µM) than *Streptomyces* sp. RSA2 (9.05 µM), which suggests slightly better inhibitory potential, *Streptomyces rochei* RSA1 also had a stronger binding affinity (B.E = -9.86 kcal/mol) than *Streptomyces* sp. RSA2 (-9.23 kcal/mol), indicating a tighter ligand interaction. The two species displayed roughly the same binding energy. Additionally, the *Streptomyces rochei* RSA1 exhibits lower binding distances (2.64, 2.52 Å) than the *Streptomyces* sp. RSA2 (2.98 Å), suggesting that the ligand is closer to important residues, which probably contributes to its higher binding energy. Additionally, their interaction with amino acid residues revealed that *Streptomyces rochei* RSA1: binds via hydrophobic LeuA158 with electrostatic contacts with polar amino acids (LysA105, ArgA157, ArgA54, AspA160, and AspA66). The polar amino acids Arg150, Asp153, Asp155, and the hydrophobic Phe156 are all in contact with Streptomyces sp. RSA2. *Streptomyces rochei* RSA1 van der Waals + H-bond + Desolation (vdW) showed greater non-covalent interactions with a value of −22.7 kcal/mol, which is more favorable *Streptomyces* sp. RSA2 −21.92 kcal/mol. Both exhibit noteworthy electrostatic contributions (*Streptomyces rochei* RSA1: −13.64 kcal/mol; *Streptomyces* sp. RSA2: −13.07 kcal/mol); nevertheless, *Streptomyces rochei* RSA1 greater value corresponds to its better affinity, and both their RMSD and total internal energy are similar at about − 22 kcal/mol, indicating high docking dependability with PDBID: 1O9G^29–31^. Thus, we deduced that LeuA158 has a hydrophobic interaction, AspA160/AspA66 demonstrated intramolecular interaction, and *Streptomyces rochei* RSA1 had LysA105 & ArgA157/ArgA54 with positive charges, which is most likely for hydrogen bond interaction between them. Pro192 displayed a stiff loop conformation to place additional residues. while, *Streptomyces* sp. RSA2 displayed Arg150/Asp153/Asp155 with a negative charge pocket that binds with cationic cavities inside the ligand. Therefore, *Streptomyces rochei* RSA1 binding is more electrostatic, whereas *Streptomyces* sp. RSA2 might depend on a balance between hydrophobic and polar forces.

**Table 10:** Docking analysis of *Streptomyces rochei* RSA1 and *Streptomyces* sp. RSA2 with PDBID:1O9G, 1J5E, and 9QT5 :

Protein	Binding Energy (B.E)	Binding distance	Inhibitory constant, Ki (uM)	Binding amino acids	vdW + H bond + desolv Energy	Electrostatic energy	Total Internal, Unbound Energy	RMSD
***PDBID:1O9G***								
***Streptomyces rochei MK166029***	−9.86	2.64, 2.52	9.65	LysA105, Arg A157, Leu A158, Arg A54, Asp A160, Asp A66	−22.7	−13.64	−22.93	0.94
***Streptomyces sp. MK166030***	−9.23	2.98	9.05	Pro 192, Arg 150, Asp 153, Phe 156, Asp 155, Asp 153	−21.92	−13.07	−22.02	0.90
***PDBID:1J5E***								
***Streptomyces rochei MK166029***	−12.92	2.49	8.32	Lys A116, Leu A1a55, Arg 54, Asp A56, Asp 166	−18.02	−9.02	−19.02	0.91
***Streptomyces sp. MK166030***	−11.73	2.82	9.01	Asn A104, Arg 95, Leu A98, Gly A99	−17.92	−8.62	−17.3	0.98
***PDBID***:*9QT5*								
***Streptomyces rochei MK166029***	−7.98	2.6, 2.71	5.87	Lys 124, His 123, Leu 125, Arg 55	−14.98	−9.64	−13.64	0.93
***Streptomyces sp. MK166030***	−7.02	3.22	6.10	Lys 128, Cys 129, Leu 125, His 125, Lys 124	−13.45	−8.26	−11.94	1.01

Additionally, the dynamic analysis trajectories revealed that *Streptomyces rochei* RSA1 forms the more stable complex, with a lower average RMSD and less flexibility, hovering between 0.13 and 0.17 nm (average ~ 0.155 nm), while *Streptomyces* sp. RSA2 stays between 0.16 and 0.21 nm (average ~ 0.185 nm), which is moderately stable but shows higher mobility, potentially reflecting weaker or more dynamic interactions. With its biggest peaks around residues ~ 70–80 and ~ 130–140, RMSF revealed that *Streptomyces rochei* RSA1 (gold) primarily stays between ~ 0.06–0.11 nm, reflecting greater stiffness that is consistent with its increased binding energy. Also, Streptomyces sp. RSA2 (orange dashed) fluctuates higher overall (~ 0.08–0.13 nm), with prominent peaks near residues ~ 20–30, ~ 50–60, and ~ 100-110 with more flexibility, especially at specific loop regions. Its RMSF Heatmap displayed uniform bands and horizontal lines with comparable flexibility and RMSF hotspots. While, *Streptomyces rochei* RSA1 majority of rows are cooler (purple–teal), with only sporadic yellow bursts around residues ~ 70–80 and ~ 130–140. In contrast, *Streptomyces* sp. RSA2 shifts toward green–yellow nearly throughout, highlighting broad regions of enhanced mobility^32,33^.

Furthermore, distinct interactions with *Streptomyces rochei* RSA1 and *Streptomyces* sp. RSA2 was observed when various ribosomal subunits, such as 30 s and 50 s ribosomes, interacted with each other. *Streptomyces* rochei’s PDBID:1J5E binding energy interacts with Lys A116, Leu A55, Arg 54, Asp A56, and Asp 166 (polar + charged residues), while *Streptomyces* sp. RSA2 binds via Asn A104, Arg 95, Leu A98, and Gly A99 (mixed polar/nonpolar). Its inhibitory constant is 8.32 µM, which is lower than *Streptomyces* sp. RSA2 (Ki = 9.01 µM).

*Streptomyces rochei* RSA1 had two near binding distances (2.6 Å, 2.71 Å); however, *Streptomyces* sp. RSA2 had a greater distance (3.22 Å), indicating poorer binding, according to the PDBID:9QT5 interaction with lower binding with − 7.98 kcal/mol and − 7.02 kcal/mol. Additionally, their relationship with amino acids demonstrated that *Streptomyces* sp. RSA2 has additional interactions with Cys 129 and His 125, which may not be as effective in contributing to binding energy; both interact with Lys 124 and Leu 125. Their energy of van der Waals plus H-bond plus desolvation. Its electrostatic energy with − 9.02 kcal/mol in 1J5E for *Streptomyces rochei* RSA1 indicates that salt bridges and charged interactions (like Lys-Arg-Asp) help stabilize binding, and its total energy showed higher, more negative in for *Streptomyces rochei* RSA1, indicating a more stable bound conformation. *Streptomyces rochei* in 9QT5 has the highest electrostatic contribution (−9.64), possibly due to His 123 and Lys 124 interactions. This suggests that hydrophobic packing and hydrogen bonding drive binding in both cases. Better structural compatibility is implied by the lower RMSD values of both *Streptomyces rochei* RSA1 (0.91 Å in 1J5E, 0.93 Å in 9QT5) compared to *Streptomyces* sp. RSA2 Therefore, we deduced that 9QT5 lacks strong ionic couples, which results in weaker binding, whereas 1J5E has more ideal residue chemistry (charged + hydrophobic balance).

Both trajectories rise rapidly from ~ 0.14 nm to ~ 0.17 nm within the first 20 ns, indicating equilibration, according to PDBID: 1J5E. After ~ 20 ns, they plateau with fluctuations of ± 0.01–0.02 nm, indicating stable sampling around their average conformations, with the blue trace (*S. rochei*) settling slightly lower on average than the red (S. sp.), suggesting slightly tighter backbone packing in the *S. rochei* complex. Both strains share low mobility core regions (RMSF ~ 0.05–0.07 nm) that correspond to structured helices/strands, as well as high mobility loops (peaks up to ~ 0.10–0.12 nm). The RMSF revealed red bars for *Streptomyces rochei* RSA1 and blue bars for *Streptomyces rochei* RSA1. Red peaks slightly dominate at residues ~ 20–30 and ~ 100–110, indicating that *Streptomyces rochei* RSA1 loops are more labile there, while *Streptomyces rochei* RSA1 exhibits sharper spikes around ~ 70–80 and ~ 130–140. As loops and side chains rearrange, the H-bond occupancy fluctuates between 50 and 55 hydrogen bonds, and the *Streptomyces rochei* RSA1 (blue) retains somewhat higher counts at specific times (e.g., 40–60 ns). It’s dynamic cross correlation (DCC) Heatmap, which displayed pairwise residue motion correlations (blue = anti-phase, red = in-phase). The blue patches identify regions moving oppositely, possibly allosteric segments, while the strong red diagonal verifies each residue’s perfect self-correlation with two helices moving together. Additionally, the RMSD of both curves in the PDBID:9QT5 System Analysis increases from around 0.14 nm to approximately 0.17 nm in about 20 ns, and the green trace of *Streptomyces rochei* RSA1 consistently stays marginally below the orange (S. sp.) trace, suggesting a slight stability advantage for *Streptomyces rochei* RSA1 in this complex. S. sp. (orange) had higher fluctuations around residues ~ 50–60 and ~ 120–130, according to the RMSF.

Whereas, *Streptomyces rochei* RSA1 (green) displays its highest mobility near ~ 10–20 and ~ 80–90, and its Hydrogen bond and oscillation between ~ 45 and ~ 57 bonds and its S. sp. (orange) often edges above *Streptomyces rochei* RSA1 (green) in bond number around 0–20 ns and 60–80 ns, suggesting dynamic windows where *Streptomyces* sp. RSA2 makes more transient contacts.

## Discussion

Endophytic actinobacteria, particularly *Streptomyces* dwelling internally in the host plants’ tissues, are gaining significant focus owing to their efficiency to set off large array of secondary metabolites having vast biological activities and functions^34^. Out of 18 endophytic *Streptomyces*spp. recovered from 16 root samples, 8 isolates revealed potency against the tested bacterial pathogens. Likewise^35,36^, disclosed that actinobacteria, *Streptomyces* play a significant function in the plant rhizosphere by producing a wide array of antibacterial compounds. *Streptomyces* are distinguished by many mechanisms of action, including competition, antibiotic synthesis, and their role as potent biocontrol agents.

The isolates RSA1 and RSA2 displayed the uppermost antagonistic features against bacteria, they were identified as *Streptomyces rochei* RSA1 and *Streptomyces* sp. RSA2, respectively. The antibacterial potency of their extracts revealed higher efficiency against bacteria in comparison with 6 commercial antibiotics. Such findings come in accordance with Pazhanimurugan et al.^37^, who depicted that the compounds recovered from *Streptomyces rochei* M32 suppressed broad-spectrum of drug resistant pathogens and enteric pathogens. Additionally, Al-Ansari et al.^38^, illustrated that *Streptomyces* sp. ES2 extract has shown significant efficacy against both Gram-positive and Gram-negative bacteria. This is consistent with findings by Malash et al.^39^, who illustrated that the *Streptomyces* sp. MMM2 extract performed higher efficacy towards pathogenic bacteria in comparison with various commercial antibiotics.

GC-MS analysis illustrated production of 3 bioactive compounds by the two isolates *Streptomyces rochei* RSA1 and *Streptomyces* sp. RSA2: Pyrimidine- 4, 6(3 H 5 H)- dione; phenol, 2,4-bis(1,1-dimethylethyl) and 1,3-cyclohexanedione, 2, 5, 5-trimethyl.

Pyrimidine refers to a class of chemical compounds distinguished by two carbonyl groups substituting a pyrimidine ring. Pyrimidines exhibit therapeutic applications, including antibacterial including tuberculosis, antiviral including HIV, anticancer, antihypertensive, hypoglycaemic, anticonvulsive, anti-inflammatory, and analgesic properties^40,41^. They are of paramount significance due to their non-toxic nature^42^.

2,4-bis (1,1-dimethylethyl) is considered an ancestor to many compounds, it is also broadly used as antioxidants and UV stabilizers or light protection agents^43^. It could also be isolated from sweet potato, avocado and pomegranate root extracts^44,45^ and also as metabolic compounds of *Streptomyces* sp. RSA2, *Bacillus subtilis* and *Vibrio *sp. ^46,47^. Furthermore, a significant antibacterial influence of *Bacillus zhangzhouensis* OMER4, whose extract comprised phenol and 2, 4-bis (1, 1-dimethyl ethyl) as a principal ingredient. The antioxidant and neuroprotective properties of phenol, 2,4-bis (1,1-dimethylethyl) has been reported. The inhibition of biofilm development in the uropathogen *Serratia marcescens* has been documented^48^. Furthermore, the antibiofilm efficacy of 2,4-bis (1,1-dimethylethyl) extracted from *B. subtilis* linked with the surface of the seaweed *Gracilaria gracilis* affected *Streptococcus pyogenes*, a β-hemolytic *Streptococcus* of group A^49^.

1,3-Cyclohexanedione and its derivatives are regarded as essential for the synthesis of many chemical compounds that exhibit antibacterial properties such as xanthene^50^ as well as anti-inflammatory properties, anticancer, and antiviral influence. Pyran, a derivative of 1,3-cyclohexanedione, possesses biological and pharmaceutical potential, including diuretic, anticoagulant, anticancer, and spasmolytic effects^51^. Oxime ethers exhibit notable anti-plasmodial and powerful fungicidal properties^52^.

Piperidine, 1, 6-phenazinediol, benzenepropanoic acid, and bis (2-ethylhexyl) ester are the four other bioactive substances that were isolated from *Streptmyces rochei* RSA1. Hexanedioic acid, or bis-(2-ethylhexyl) ester-, was extracted from *Streptmyces rochei* RSA1. An industrial chemical, this molecule is utilized in a variety of items, including cosmetics^53^. Additionally, hexanedioic acid was generated from *Streptomyces* sp. TN262 extract, it displayed antagonistic attitude against *Micrococcus luteus* LB *Staphylococcus aureus* ATCC 6538, 14,110, *Escherichia coli* ATCC 8739 *Salmonella enterica* ATCC43972, and *Fusarium* sp^54^. Hexanedioic acid’s antibacterial properties towards methicillin-resistant *Staphylococcus aureus* (MRSA). Bis-(2-ethylhexyl) ester- (hexanedioic acid) made up 1.36% of the extract of cocoyam (*Colocasia esculenta*), which is known for its anti-inflammatory, anti-cancer, and antioxidant qualities^55^. According to reports, benzoenepropanoic works well against a variation of Gram-negative bacteria, for example: *Pseudomonas aeruginosa*, *Escherichia coli* and *Klebsiella pneumonia*, in addition to several fungi as *Candida albicans*^56^. It is well known that benzoenepropanoic acid has antifungal and antioxidant properties^57^. Furthermore, benzenepropanoic acid is identified as a major ingredient of *Woodfordia fruticosa* leaves extract via GC-MS analysis. utilized as spices, flavorings, sweeteners, fragrances, emulsifiers, and preservatives, and they preserve the original ingredients in food items. They are also constituents of cosmetic goods such as detergents, fabric softeners and perfumes^58^.

Piperidine and its derivatives are present in minimal quantities in tobacco and black pepper unlike *Psyclocoulon absimile* which contains higher amounts^59^. Piperidine moiety- containing compounds exhibit a wide array of actions, including antibacterial, anticancer, analgesic, anti-inflammatory, and antipsychotic properties, establishing piperidine as a crucial foundation in pharmaceutical development^60,61^. Medications containing piperidine are effective in treating diabetes and schizophrenia^62^.

5-octadecane and cyclo (-pro-phe) were recorded from of *Streptomyces* sp. RSA2 extract. Studies demonstrate that 5-octadecene was also identified from extracts of mangrove originated *Streptomyces cheonanensis* VUK-A extract, it affects potently on agriculturally and medicinally important fungi and bacteria. Terrestrial *Streptomyces* sp. TN 272 was also reported for production of 5-Octadecene^63^. Antimicrobial activity 5-octadecene was also demonstrated^64,65^. Cyclo (-pro-phe) was synthesized from 0.05 of *Streptomyces* sp. RSA2 extract. Cyclo (-pro-phe) was a constituent of the crude extract of endophytic *Streptomyces* SUK 25, exhibiting significant bioactivity against *Staphylococcus aureus*, *Enterococcus raffinosus*, *Pseudomonas aeruginosa*, *Acinetobacter baumanii*, *Klebsiella pneumoniae* and *Enterobacter* spp^66^. This chemical is notable for its extensive array of biological actions, including antiviral, antibacterial, antifungal, anti-tumor and inhibition of quorum-sensing characteristics^67–71^.

## Conclusion

This research shows that endophytic Streptomyces spp., which were isolated from Arachis hypogaea roots, are an excellent source of anti-bacterial metabolites. Of the eighteen isolates, *Streptomyces rochei* RSA1 (MK166029) and *Streptomyces* sp. RSA2 (MK166030) exhibited the best inhibition of both Gram-positive and Gram-negative bacterial pathogenic, as well as better inhibition than some commercial antibiotics. The analysis of their extracts with GC–MS and FT-IR indicated these extracts were dominated by 2-(butylthio)pyrimidine-4,6(1 H,5 H)-dione, along with various phenolic, diketone, and cyclic dipeptide compounds with various biological activities. Further, molecular docking and molecular dynamics simulations showed strong and stable binding of the ligands to key bacterial enzymes and ribosomal subunits, suggestive of their potential as antimicrobial candidates. So we concluded that these results emphasize the therapeutic potential of peanut-derived Streptomyces as natural sources for new antibacterial agents. Future studies should focus on the purification of the major metabolites, deeper mechanistic studies, and pharmacological and toxicological assessments in vivo for their eventual conversion into antibiotic scaffolds.

## Data Availability

The data that support the findings of this study are available from the corresponding author upon reasonable request.
